# *De novo* assembly and annotation of the Zhe-Maidong (*Ophiopogon japonicus* (L.f.) Ker-Gawl) transcriptome in different growth stages

**DOI:** 10.1038/s41598-017-03937-w

**Published:** 2017-06-15

**Authors:** Huijun Liu, Ying Wang, Tingzhang Wang, Xuhui Ying, Rongrong Wu, Huan Chen

**Affiliations:** 1 College of Pharmaceutical Science, Zhejiang University of Technology, Hangzhou, 310014 China; 2 Zhejiang Institute of Microbiology, Hangzhou, 310012 China; 3 Key laboratory of microbial technology and bioinformatics of Zhejiang Province, Hangzhou, 310012 China; 4 Department of Cardiology, Second Affiliated Hospital, College of Medicine, Zhejiang University, Hangzhou, 310009 China; 5 Cardiovascular Key Laboratory of Zhejiang Province, Hangzhou, 310009 China; 6 ChiaTaiQingchunbao Pharmaceutical Co., Ltd, Hangzhou 310012, China

## Abstract

Zhe-Maidong (*Ophiopogon japonicus* (L.f.) Ker-Gawl) is a traditional medicinal herb in the family *Liliaceae* that has significant pharmacological effects on immunity and cardiovascular disease. In this study, three different growth stages of Zhe-Maidong were investigated using RNA-seq, and a total of 16.4 Gb of raw data was obtained. After filtering and assembling, 96,738 unigenes with an average length of 605.3 bp were ultimately generated. A total of 77,300 unigenes were annotated using information from five databases, including the NT, NR, SwissProt, Kyoto Encyclopedia of Genes and Genomes (KEGG) and Gene Ontology (GO) databases. Additionally, the mechanisms of flavonoid, saponin and polysaccharide biosynthesis and of accumulation at different stages of tuber development were also characterized. From the first to third years, the contents of flavonoids, saponins and polysaccharides all increased, whereas the expression levels of related genes decreased in the flavonoid and saponin pathways and first increased and then decreased in the polysaccharide pathway. The results of this study provide the most comprehensive expressed sequence resource for Zhe-Maidong and will expand the available *O*. *japonicus* gene library and facilitate further genome-wide research and analyses of this species.

## Introduction

Zhe-Maidong (ZMD), also known as *Ophiopogon japonicus* (L.f.) Ker-Gawl, is an evergreen perennial in the *Liliaceae* family and a famous Chinese traditional medicinal herb. ZMD is planted primarily in the cities of Cixi and Hangzhou in Zhejiang Province, China^1^. Modern pharmacological studies show that the tubers of *O*. *japonicus* are rich in flavonoids, saponins and polysaccharides, which have beneficial effects on immunomodulatory^2^, cardioprotective^3^, neuroprotective^3^, antimicrobial^3^, antihyperlipidaemia^4^, antioxidant^5^, anticancer^6^, anti-inflammation^7^ and antidiabetic^8^ activities. To date, more than 30 flavonoids^8^, 70 saponins^9^ and 10 bioactive polysaccharides^3^ have been isolated from the tubers of *O*. *japonicus*. ZMD is one of two basic ingredients in Shenmai injections and one of the most widely used herbal medicines in traditional Chinese medicine (TCM). ZMD is frequently used to treat atherosclerotic coronary heart disease and viral myocarditis or is co-administered with other prescribed medicines in certain circumstances as an organ protector^10^. The tuber is the storage organ of *O*. *japonicus* and is derived from modified roots. Tuber development is a complex process and depends on the balanced expression of genes controlled by environmental and endogenous factors^11^. However, little is known about the mechanisms that regulate tuber growth and various changes in metabolites.

The transcriptome is the complete set and quantity of transcripts in a cell at a specific developmental stage and provides information on gene expression and gene regulation related to primary or secondary metabolite biosynthesis^12, 13^. As a result of the rapid development of next generation sequencing, RNA sequencing (RNA-seq) is a useful method for studying the metabolic pathways of medicinal components and related gene expression in different samples or tissues, such as flavonoid biosynthesis in *Carthamus tinctorius* ^14^, *Pueraria lobata* ^15^ and *Polygonum cuspidatum* ^16^; saponin biosynthesis in *Asparagus racemosus* ^17^, *Panax japonicus* ^18^, *Panax notoginseng* ^19^ and *Panax quinquefolius* ^20^; glycyrrhizin biosynthesis in *Glycyrrhiza uralensis* ^21^; and fatty acid biosynthesis in *Eucommia ulmoides* ^22^. With this method, related gene expression across samples or tissues can also be analyzed. For ZMD, genomic and transcriptomic studies have not yet been reported; only a study discriminating *O*. *japonicus* and *Liriope spicata* (“shanmaidong”) has been published, which used DNA sequences^23, 24^ and EST-SSR markers^25^ or SCAR markers^26^ to study molecular diversity in the two species. ZMD usually requires three years from planting to harvest; according to the history of planting, ZMD tubers will gradually decay and disappear after three years. Because of this unique characteristic, RNA-seq was used to study the differential expression of genes in three different growth stages.

In this study, ZMD tubers during three different growth stages (one year old: Y1, two years old: Y2, three years old: Y3) were collected and sequenced using the Illumina HiSeq-2000 platform, and a total of 16.4 Gb of raw data was obtained. The assembled unigenes were annotated against public databases using NCBI non-redundant nucleotide sequence (NT), NCBI non-redundant protein (NR), SwissProt, Gene Ontology (GO) and Kyoto Encyclopedia of Genes and Genomes (KEGG) classifications. After assembly and annotation, the different expression levels of genes for the three different years were identified. We also identified the pathways of flavonoid, saponin and polysaccharide biosynthesis and analysed the expression of related genes. Additionally, the contents of methylophiopogonanone A, total flavonoids, total saponins and total polysaccharides were quantified using high-performance liquid chromatography (HPLC) and spectrophotometric analysis (SP). Furthermore, variation in the transcriptome was investigated during the three different growth stages, and comparative analyses revealed that the studied pathways were significantly affected. Therefore, the results of this study can serve as a guide for the development of breeding strategies.

## Results and Discussion

### Illumina sequencing and *de novo* assembly

**Figure 1:**
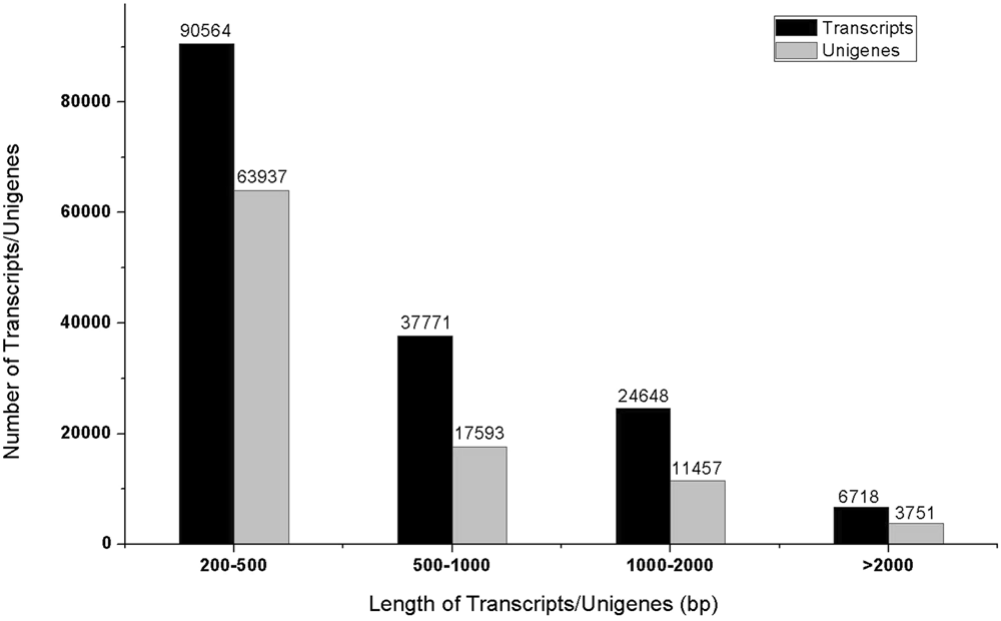
Length distribution of assembled transcripts/unigenes obtained from high-quality clean reads. The lengths range from 200 bp to more than 2,000 bp. Black bar, transcripts; grey bar, unigenes.

To obtain a global overview of gene expression in *O*. *japonicus* tubers, three growth stages of ZMD tubers (Y1, Y2 and Y3) were collected. Total RNA was extracted, and mRNAs were purified to construct paired-end RNA-seq libraries. A total of 20,346,421 (125 × 2 base), 21,499,762 (125 × 2 base) and 24,610,833 (125 × 2 base) raw reads were obtained, which accounted for approximately 5.0, 5.3 and 6.1 Gb of sequence data, respectively. The raw data were cleaned with Trimmomatic^27^ and the pooled data were assembled with Trinity software. Ultimately, 159,701 assembled transcripts were obtained; the average length of these transcripts was 675.89 bp, and the N50 length was 961 bp (Table 1). The longest transcript of each gene was selected as the unigene, and 96,738 unigenes were generated with an average length of 605.3 bp and an N50 length of 859 bp (Table 1). The average lengths of the unigenes and N50 were close to those of *Panax japonicus* ^18^ and *Lily* ^28^ and slightly longer than those of safflower^14^, *Polygonum cuspidate* ^16^, and *Ornithogalum caudatum* ^29^. A detailed size distribution of ZMD transcripts and unigenes is shown in Fig. 1. This transcriptome database was prepared and used to identify flavonoid-, saponin- and polysaccharide-related genes and pathways.

**Table 1:** Length distributions of assembled transcripts and unigenes.

	Transcripts	Unigenes
**N50 (bp)**	961	859
**Maximum length (bp)**	10,642	10,642
**Minimum length (bp)**	224	224
**Average length (bp)**	675.89	605.3
**Total Number (>0.2 kb)**	159,701	96,738
**Total Nucleotides (bp)**	107,940,730	58,555,284

### Functional annotation and classification

**Figure 2:**
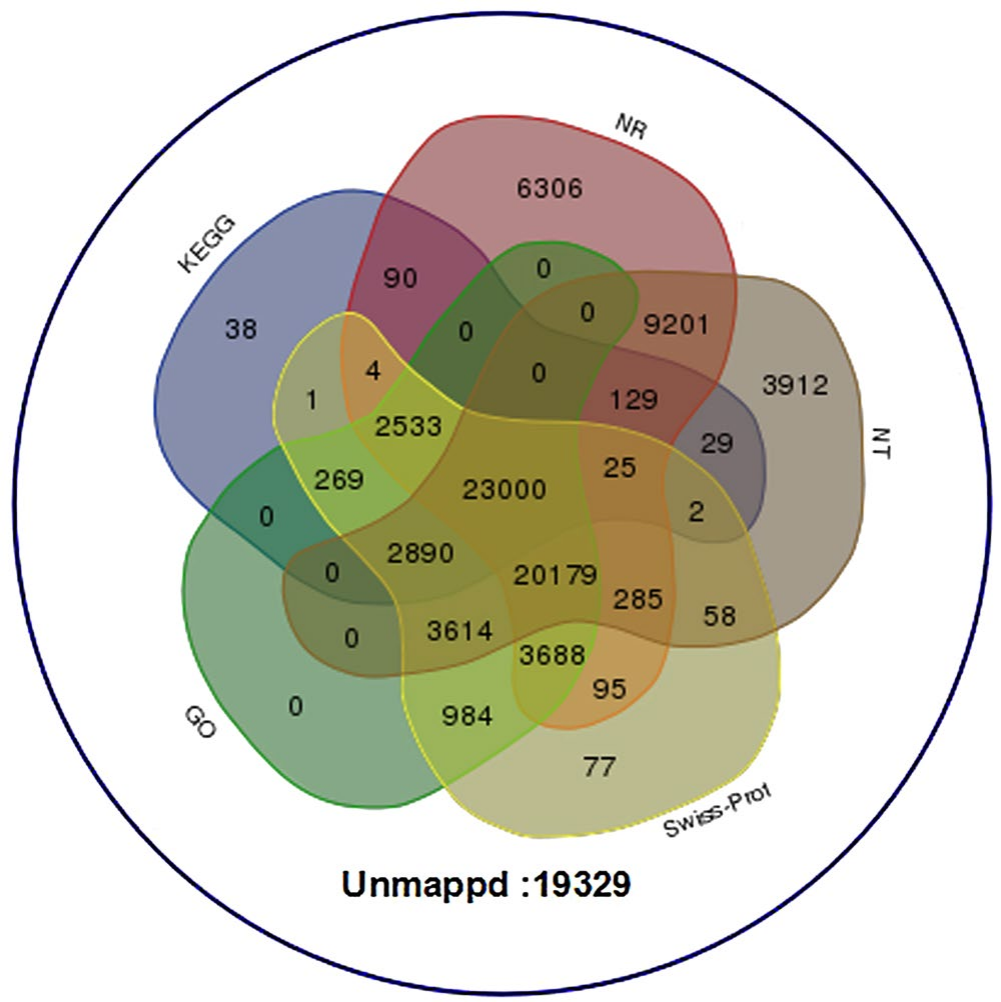
Venn diagram describing the unigenes annotated by five different database. The integration of unique similarity search results against the Nr, NT SwissProt, GO and KEGG databases. The cut-off E-value was ≤10^−5^. A total of 77,409 unigenes were grouped into five databases, and 19,329 unigenes were not found in the above databases.

To investigate their function, unigenes were annotated based on the Nr, Nt, SwissProt, GO and KEGG databases. In this study, 96,738 unigenes were searched against the five databases, and 77,409 (80.02%) unigenes were annotated according to the databases. Among them, 23,000 unigenes had hits in all five databases. Additionally, 19,329 unigenes did not significantly match the five public databases, which indicated that these unigenes might be novel transcribed sequences in *O*. *japonicas* (Fig. 2). Some unigenes were too short for statistically meaningful matches.

**Figure 3:**
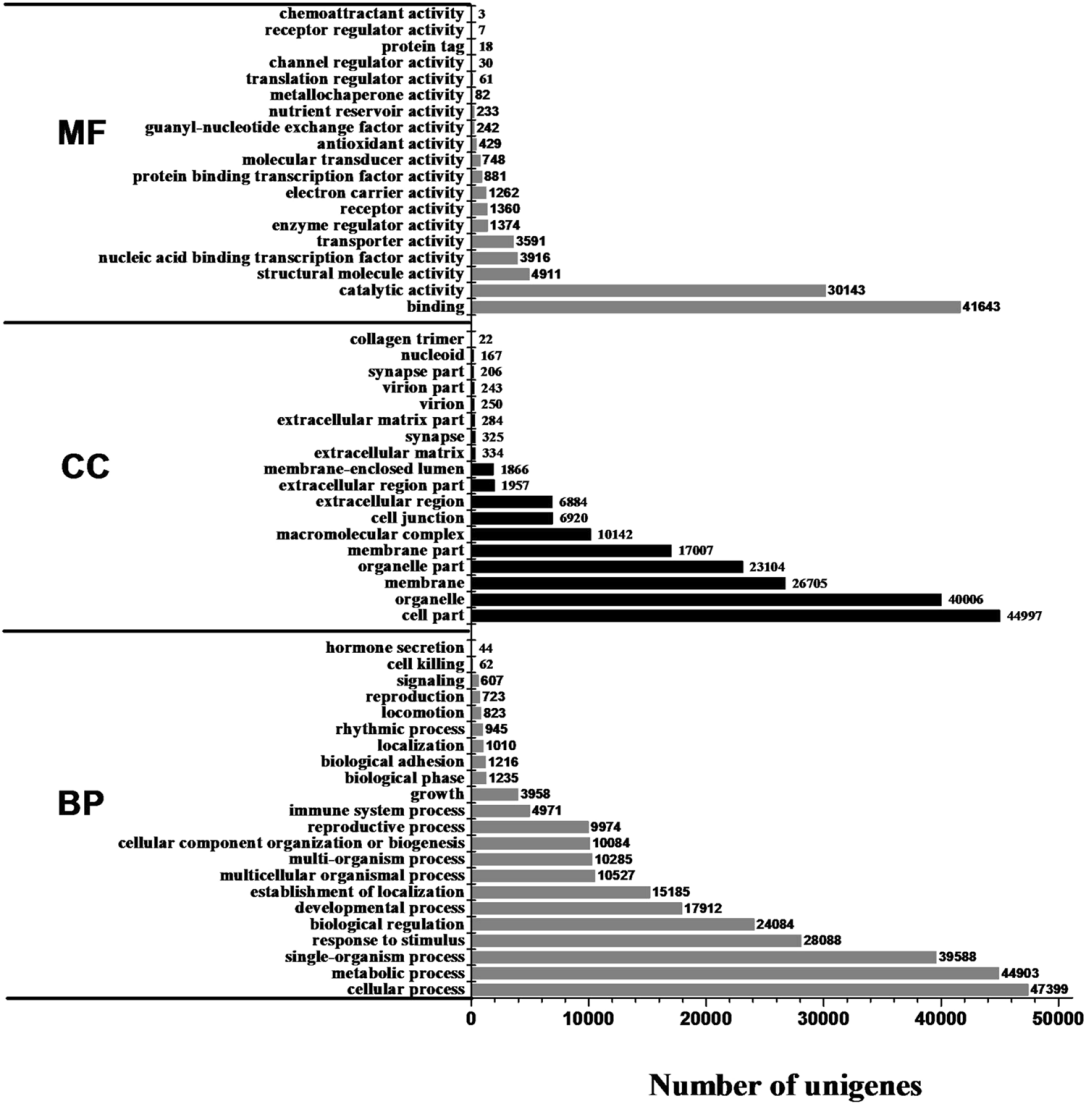
Gene ontology functional classification of assembled unigenes. Unigenes were divided into three primary categories (molecular function, cellular components and biological processes) and 59 subcategories. The y-axis represents the respective unigene categories, whereas the x-axis denotes the number of unigenes.

GO analysis showed that 57,157 unigenes (59.08%) could be categorized into 59 functional groups with 13,515 functional terms. Cellular processes (47,399, 82.8%), binding (41,643, 72.86%) and cell parts (44,997, 78.73%) were the major categories from each GO domain (Fig. 3). Three expression patterns were identified for further analysis: genes with increased pattern, genes with decreased pattern, and genes with first increased and then decreased pattern. Based on the results, most gene expression levels first increased and then decreased, and the number of genes that had reduced expression was larger than that of the genes that had increased expression. Notably, most genes with expression that decreased from the second to the third year were in the groups “cellular process”, “metabolic process”, “cell part”, “single-organism process”, “binding” and “organelle” (Figs S1, S2 and S3), which indicated that the tubers of *O*. *japonicus* gradually matured late in the second year.

**Figure 4:**
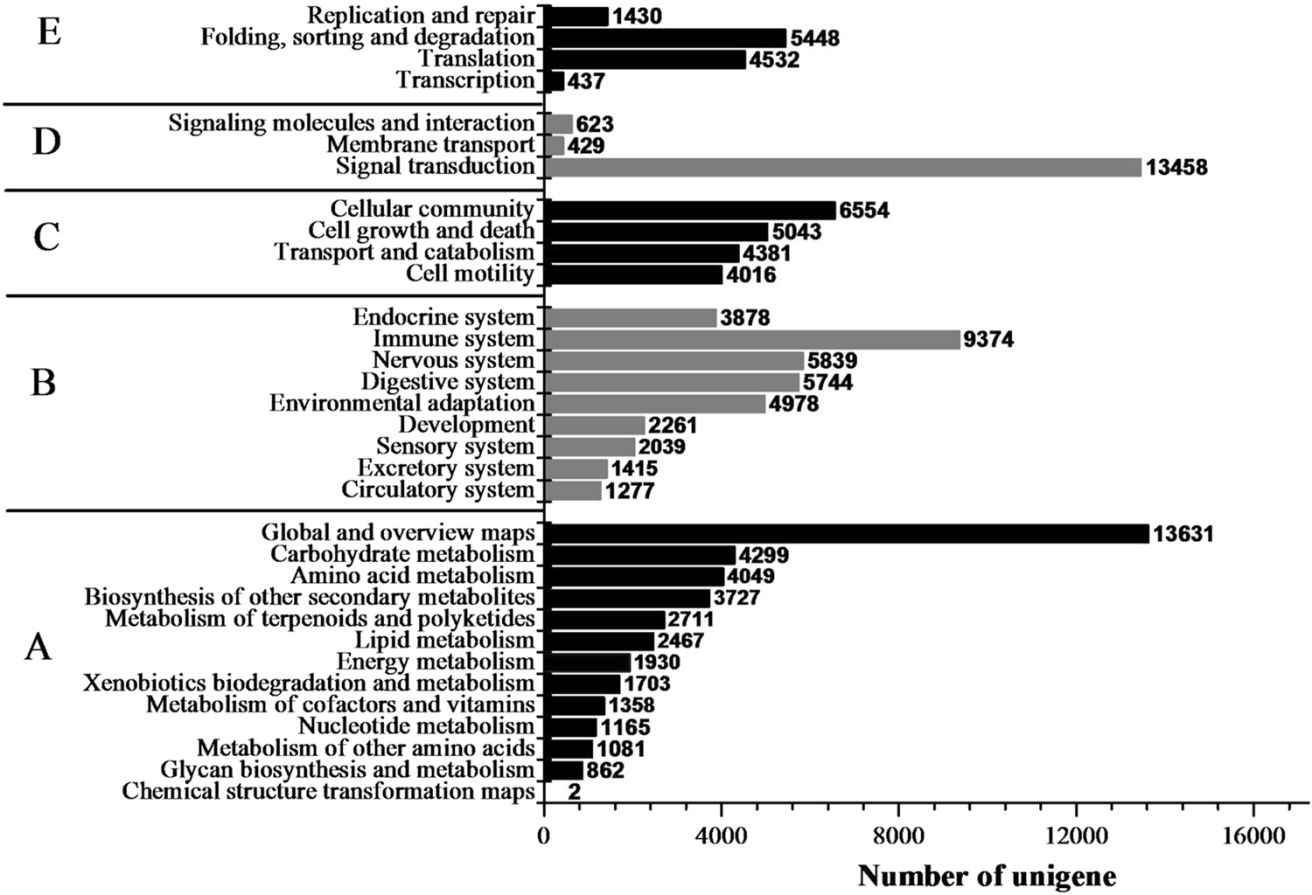
KEGG functional classification of assembled unigenes. The unigenes were divided into five primary categories (**A**) metabolism, (**B**) genetic information processing, (**C**) environmental information processing, (**D**) cellular processes and (**E**) organismal systems). The x-axis represents the number of unigenes, whereas the y-axis represents the unigene respective categories.

**Figure 5:**
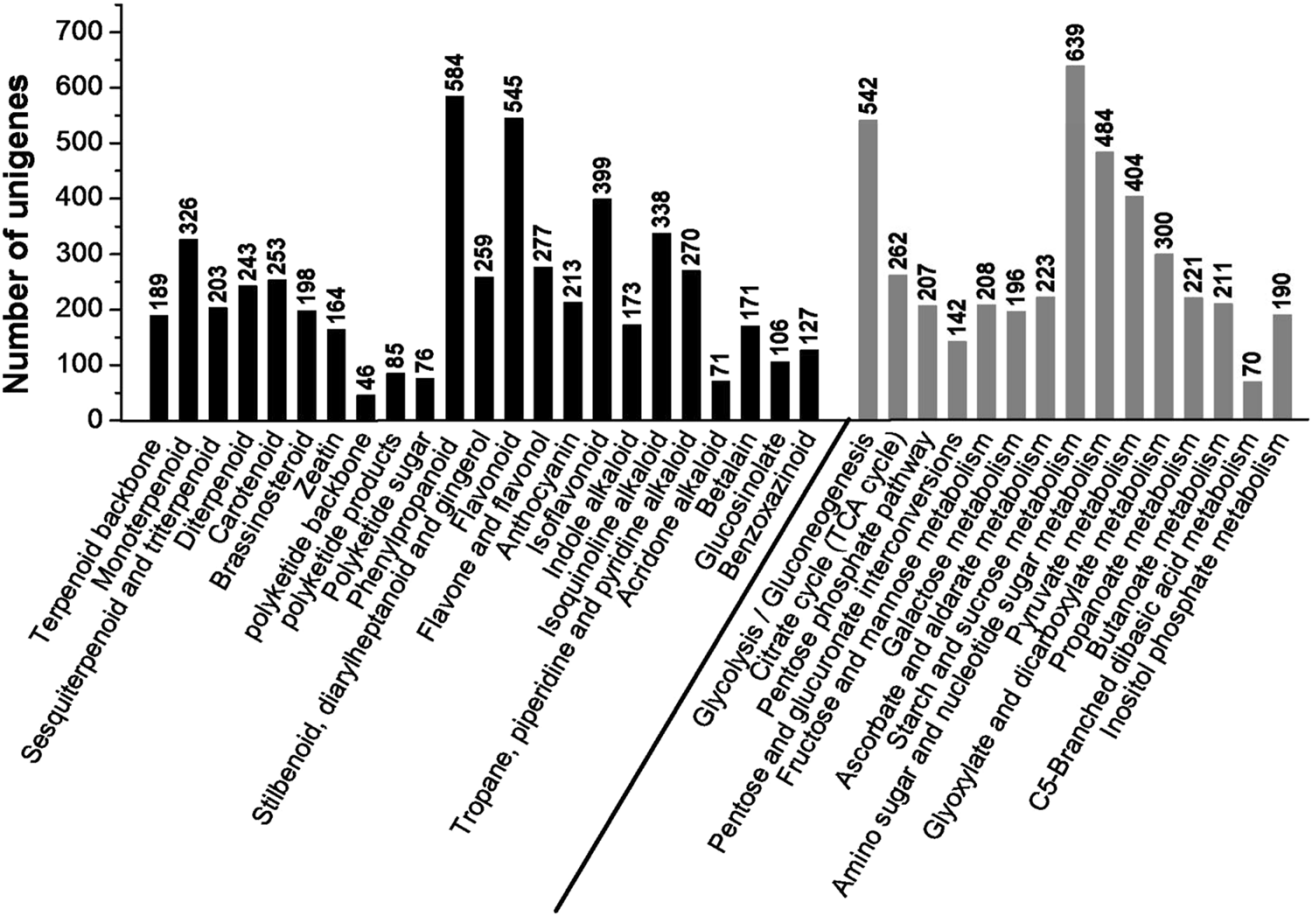
Unigenes related to secondary metabolism and carbohydrate metabolism in ZMD as annotated by the KEGG database. A total of 4488 unigenes were identified, and some genes had multiple annotated pathways. The black bars represent the secondary metabolic biosynthetic pathways, and the grey bars represent carbohydrate metabolism pathways.

The KEGG database contains data from a systematic analysis of inner-cell metabolic pathways and functions of gene products, in addition to variants specific to particular organisms^30^. Pathway-based analyses can help to further understand the biological function and expression level of genes^14^. The results showed that 29,010 (29.99%) unigenes could be assigned to five classes of 280 biological pathways according to the KEGG database (Fig. 4 and Table S1). Notably, 23 secondary metabolic biosynthetic pathways and 15 carbohydrate metabolism pathways were annotated by KEGG pathways, which included 4,488 unigenes. These are the primary metabolic pathways related to tuber development in plants and include the biosynthesis of medicinal components such as flavonoids, saponins and polysaccharides (Fig. 5). The fragments per kilobase of exon per million fragments mapped (FPKM) method was used to calculate the expression levels of these genes, and the results showed that most gene expression levels significantly decreased with planting age (Table S2).

### Identification of differentially expressed genes (DEGs) and functional annotation

**Figure 6:**
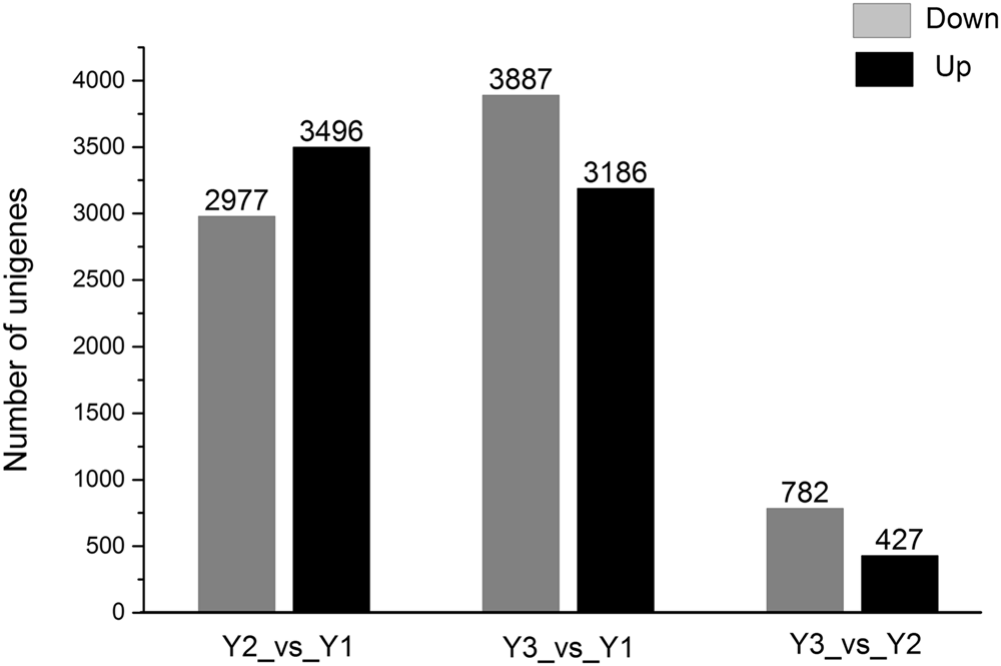
Number of DEGs at different stages of ZMD growth. Abbreviations: Y2_vs_Y1, two-year-old ZMD compared with one-year-old ZMD; Y3_vs_Y1, three-year-old ZMD compared with one-year-old ZMD; and Y3_vs_Y2, three-year-old ZMD compared with two-year-old ZMD.

To identify DEGs with different years of growth, we normalized the tag distribution for gene expression level in each library to make an effective library size and extracted the significance of differentially expressed unigenes (DEGs) with Q-values ≤ 0.05 and |log_2_-fold-change| ≥ 1 using edge-R. As shown in Fig. 6, a total of 6,473, 7,073 and 1,209 DEGs were identified in groups Y2_vs_Y1, Y3_vs_Y1 and Y3_vs_Y2, respectively (Table S3). Notably, we found that the number of DEGs in both groups Y2_vs_Y1 and Y3_vs_Y1 was significantly larger than that in Y3_vs_Y2. In this study, all DEGs were first annotated using the KEGG database. In the Y2_vs_Y1 group, of 2,977 down-regulated unigenes, 439 were related to pathways for “Biosynthesis of secondary metabolites”, and 521 were related to pathways for “Metabolic pathways”, but of 3,496 up-regulated unigenes, only 93 were related to pathways for “Biosynthesis of secondary metabolites”, and 196 were related to pathways for “Metabolic pathways” (Tables 2 and S3). We found that most unigenes related to plant metabolism decreased with years of growth in ZMD tubers.

**Table 2:** Number of DEGs related to different metabolism pathways as annotated by the KEGG database.

	Y2_vs_Y1 down	Y2_vs_Y1 up	Y3_vs_Y1 down	Y3_vs_Y1 up	Y3_vs_Y2 down	Y3_vs_Y2 up
**Biosynthesis of secondary metabolites (map01110)**	439	93	660	60	152	4
**Metabolic pathways (map01100)**	521	196	763	109	157	10

Abbreviations: Y1, one-year-old ZMD; Y2, two-year-old ZMD; Y3, three-year-old ZMD.

Additionally, all DEGs were annotated using the SwissProt database. Among them, the number of differentially expressed transcription factors (TFs) was compared at the three different stages because TFs are represented by multi-gene families and play a key regulatory role by controlling the expression of single or multiple genes through specific binding to cis-regulatory elements in the promoter regions^31^. In the Y2_vs_Y1 group, 158 genes encoded TFs representing 24 different families, whereas in the Y3_vs_Y2 group, only six genes encoded TFs representing three different families (Table S4). The TFs involved in secondary metabolite biosynthesis, such as the MYB, MADS, WRKY and bHLH families, were also represented among the DEGs in the different years of ZMD. These TFs might regulate primary or secondary metabolite biosynthesis and accumulation in ZMD at different growth stages^31^. Overall, based on the DEGs, metabolic activity gradually decreased after the second year, and the tubers of *O*. *japonicus* gradually matured late in the second year.

### Identification and expression analysis of unigenes related to flavonoid biosynthesis

**Figure 7:**
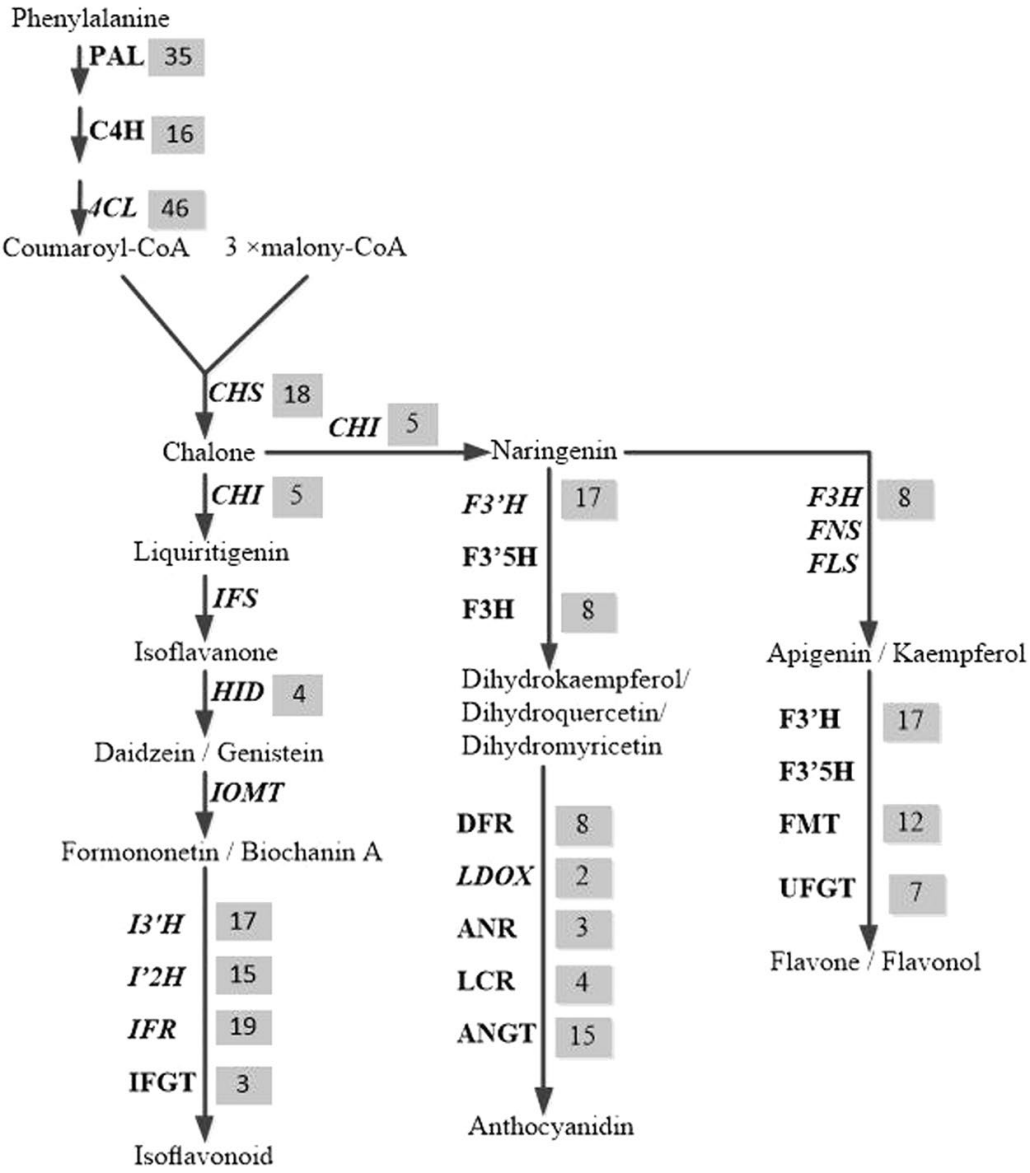
Flavonoid biosynthetic pathways in ZMD. The number of genes encoding the enzyme is showed in the grey region. Abbreviations: PAL, phenylalanine ammonia-lyase; C4H, cinnamate 4-hydroxylase; 4CL, 4-coumarate:CoA ligase; CHS, chalcone synthase; CHR, chalcone reductase; CHI, chalcone isomerase; F3H, naringenin 3-dioxygenase; FNS, flavone synthase; FLS, flavonol synthase; F′3H, flavonoid 3′-monooxygenase; F3′5H, flavonoid 3′, 5′-hydroxylase; IFS, isoflavone synthase; HID, 2-hydroxyisoflavanone dehydratase; I′2H, isoflavone 2′-hydroxylase; I′3H, isoflavone 3′-hydroxylase; IMOT, isoflavone 4′-O-methyltransferase; IFR, isoflavone reductase; IFGT, isoflavone glucosyltransferase; DFR, dihydroflavonol-4-reductase; LDOX, leucoanthocyanidin dioxygenase; LCR, leucoanthocyanidin reductase; ANR, anthocyanidin reductase; ANGT, anthocyanidin 5,3-O-glucosyltransferase; FMT, flavone methyltransferase; UFGT, UDP-glucose flavonoid glucosyltransferase.

In this study, our transcriptome revealed 254 unigenes that encode key enzymes in putative backbone biosynthesis of flavonoids from the SwissProt database. Among these unigenes, 249 and 229 were also annotated based on the KEGG and Nr databases, respectively (Table S5). The pathway of flavonoid biosynthesis is divided into two parts: the upstream pathway for naringenin or liquiritigenin biosynthesis, which includes the conversion of the aromatic amino acid L-phenylalanine (L-Phe) to naringenin/liquiritigenin under the activities of phenylalanine ammonia-lyase (PAL), cinnamate 4-hydroxylase (C4H), 4-coumarate:CoA ligase (4CL), chalcone synthase (CHS), chalcone reductase (CHR) and chalcone isomerase (CHI)^15^, and the downstream pathway, which is divided into three branches of biosynthesis: flavone/flavonol, anthocyanidin and isoflavonoid. Flavone/flavonol biosynthesis is catalysed by naringenin 3-dioxygenase (F3H), flavone synthase (FNS) and flavonol synthase (FLS) to synthesize apigenin or kaempferol, followed by further modification by hydroxylase, methyltransferase and glucosyltransferase to form flavones or flavonols^14^ (Fig. 7). Anthocyanidin is a common plant pigment, and biosynthesis from naringenin to the three structural compounds (dihydrokaempferol, dihydroquercetin, dihydromyricetin) is catalysed by flavonoid 3′-monooxygenase (F3′H), flavonoid 3′,5′-hydroxylase (F3′5H) and naringenin 3-dioxygenase (F3H) and then modified by oxygenase, reductase and glucosyltransferase to generate anthocyanidin^32^ (Fig. 7). Isoflavonoid biosynthesis is catalysed by isoflavone synthase (IFS) to synthesize isoflavanone and then modified by methyltransferase, hydroxylase, reductase and glucosyltransferase to form isoflavonoid^28^ (Fig. 7).

Flavonoids are secondary metabolites in leguminous plants, and their molecular characterization has drawn considerable interest because of their anti-cancer dietary properties and other human health benefits^33^. In this study, 120 unigenes encoded key enzymes were found in the upstream pathway, including 35 PAL, 16 C_4_H, 46 4CL, 18 CHS and 5 CHI, which are part of the precursor step for flavonoid biosynthesis, including the phenylpropanoid biosynthesis pathway that comprises a complex series of branching biochemical reactions that give rise to thousands of compounds^34, 35^. In the downstream pathway, a total of 134 unigenes were annotated: 58 unigenes for the branch for isoflavonoid biosynthesis, including 4 IFD, 15 I′2H, 17 I′3H, 19 IFR and 3 IFGT, and 76 unigenes for flavone/flavonol and anthocyanidin biosynthesis, including 17F′3H, 8 F3H, 8 DFR, 7 UFGT, 12 FMT, 4 LCR, 2 LDOX, 3 ANR and 15 ANGT (Fig. 7 and Table S5). Therefore, this research contributes to the study of valuable gene resources for the subsequent metabolic engineering of ZMD flavonoids.

**Figure 8:**
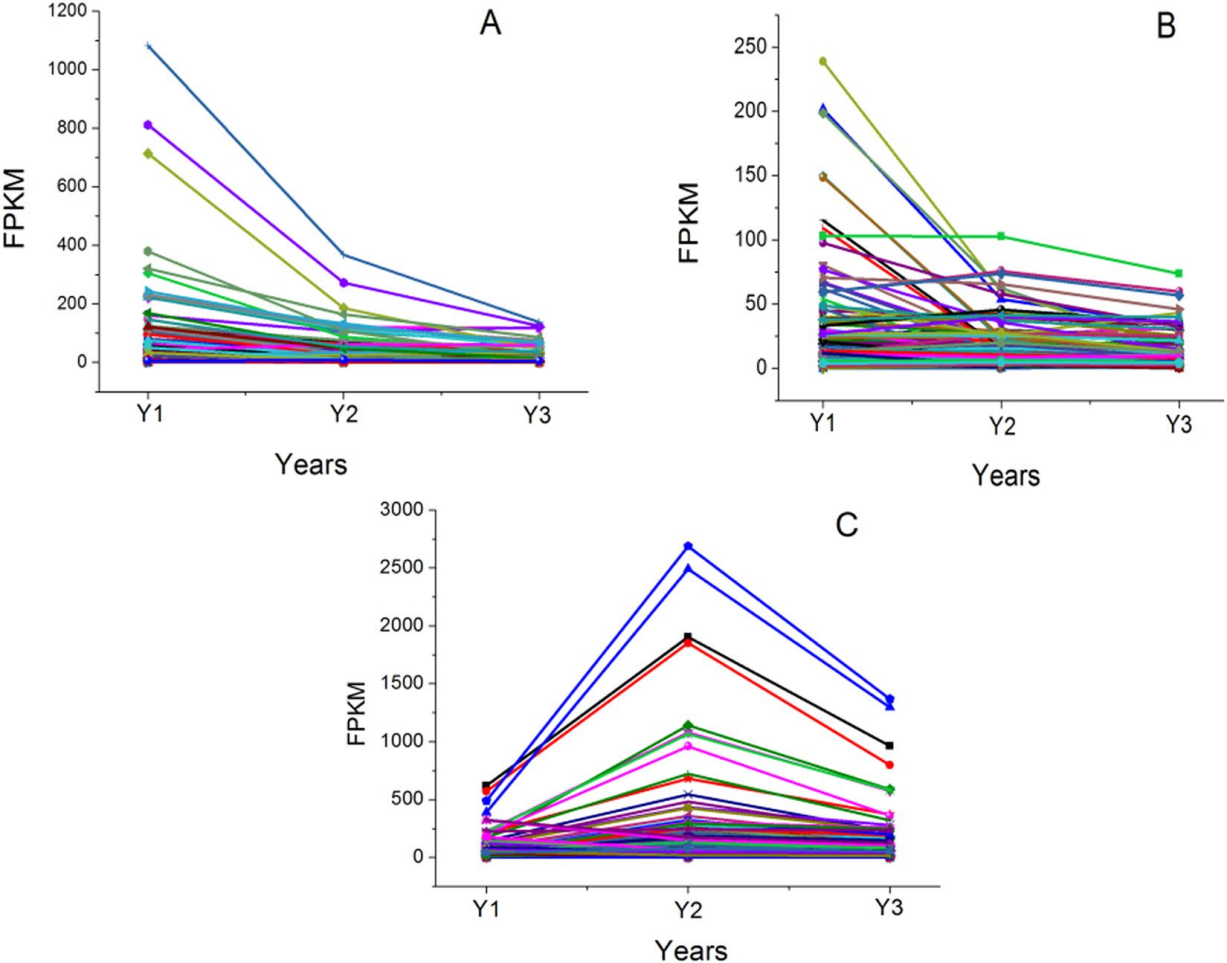
Expression patterns of the genes in the three metabolite biosynthesis pathways. (**A**): 254 unigenes are selected in the flavonoids biosynthesis; (**B**): 135 unigenes are selected in the saponins biosynthesis; (**C**): 236 unigenes are selected in the polysaccharides biosynthesis. Abbreviations: Y1, one-year-old ZMD; Y2, two-year-old ZMD; Y3, three-year-old ZMD.

**Figure 9:**
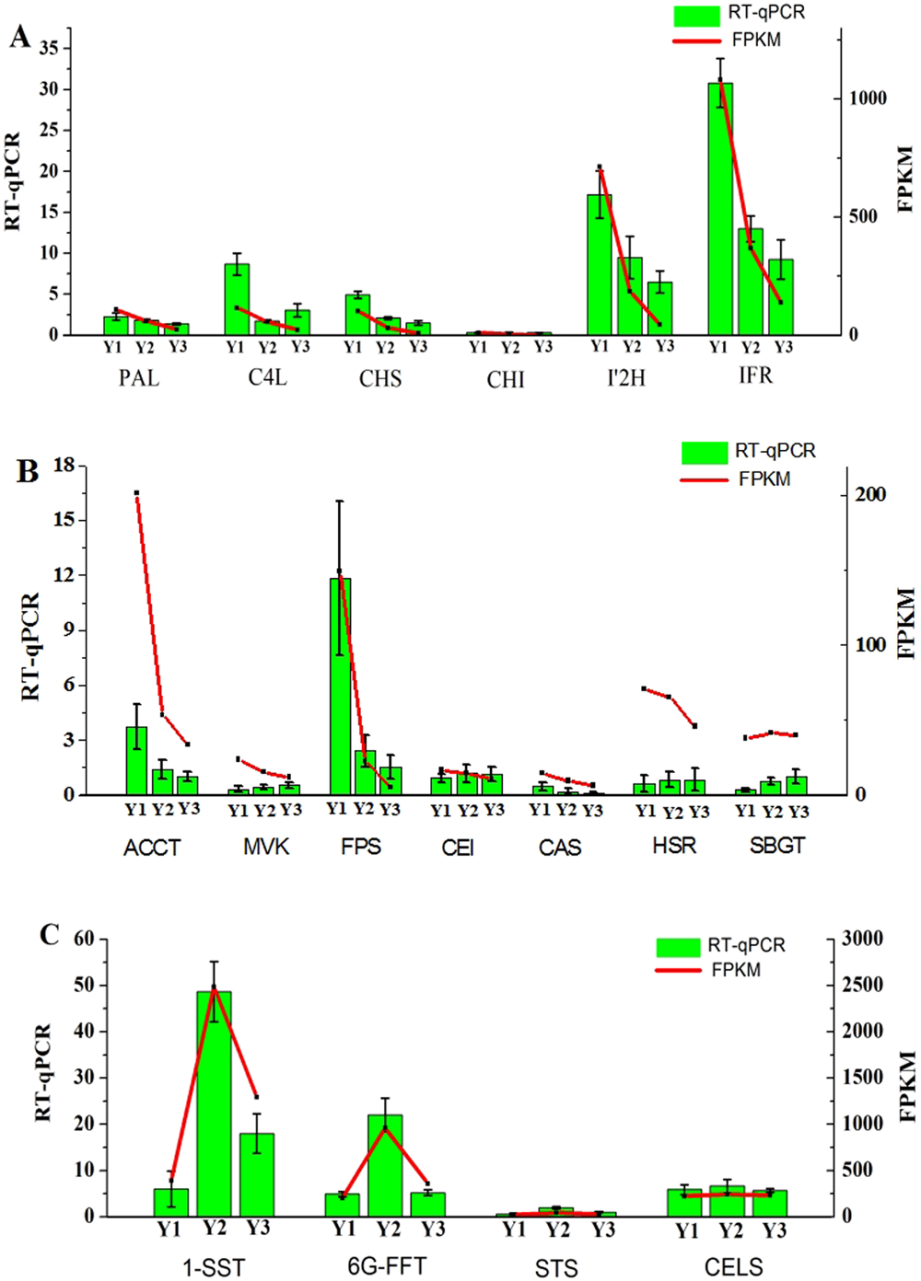
Verification of gene expression changes in different growth stage with real-time quantitative PCR. (**A**) Six representative genes are selected from flavonoids pathway; (**B**) Seven representative genes are selected from saponins pathway; (**C**) Four representative genes are selected from polysaccharides pathway. Data reported represent the average and standard error of the mean of five independent experiments. Abbreviations: Y1, one-year-old ZMD; Y2, two-year-old ZMD; Y3, three-year-old ZMD.

The FPKM method was used to calculate the expression levels of these unigenes. The results showed that the expression levels of most genes significantly decreased with planting ages (Fig. 8A), including the unigenes that encode IFR (TR22676|c0_g1, TR8005|c0_g2) and I′2H (TR7932|c3_g1, TR17129|c2_g1). The expression levels of IFR and I′2 H were relatively higher than those of other genes in Y1 and were significantly down-regulated with increasing growth stage (Table S5). IFR acts specifically on CH-CH groups with NAD^+^ or NADP^+^ as an acceptor^36^, and I′2H acts on paired donors with O_2_ as the oxidant and the incorporation or reduction of oxygen^37^. IFR and I′2H are in the family of oxidoreductases and are two typical enzymes for the biosynthetic modification of the isoflavone skeleton. To demonstrate that the unigenes from sequencing and computational analysis were indeed expressed, we also analysed the difference in gene expression profiles in Y1 and Y3 tubers. Six unigenes related to flavonoid biosynthesis (including TR7932|c3_g1 and TR22676|c0_g1) were selected for validation using quantitative RT-PCR, and the results were consistent with the FPKM values from the expression trends (Fig. 9A and Table S6).

### Identification and expression analysis of unigenes related to saponin biosynthesis

**Figure 10:**
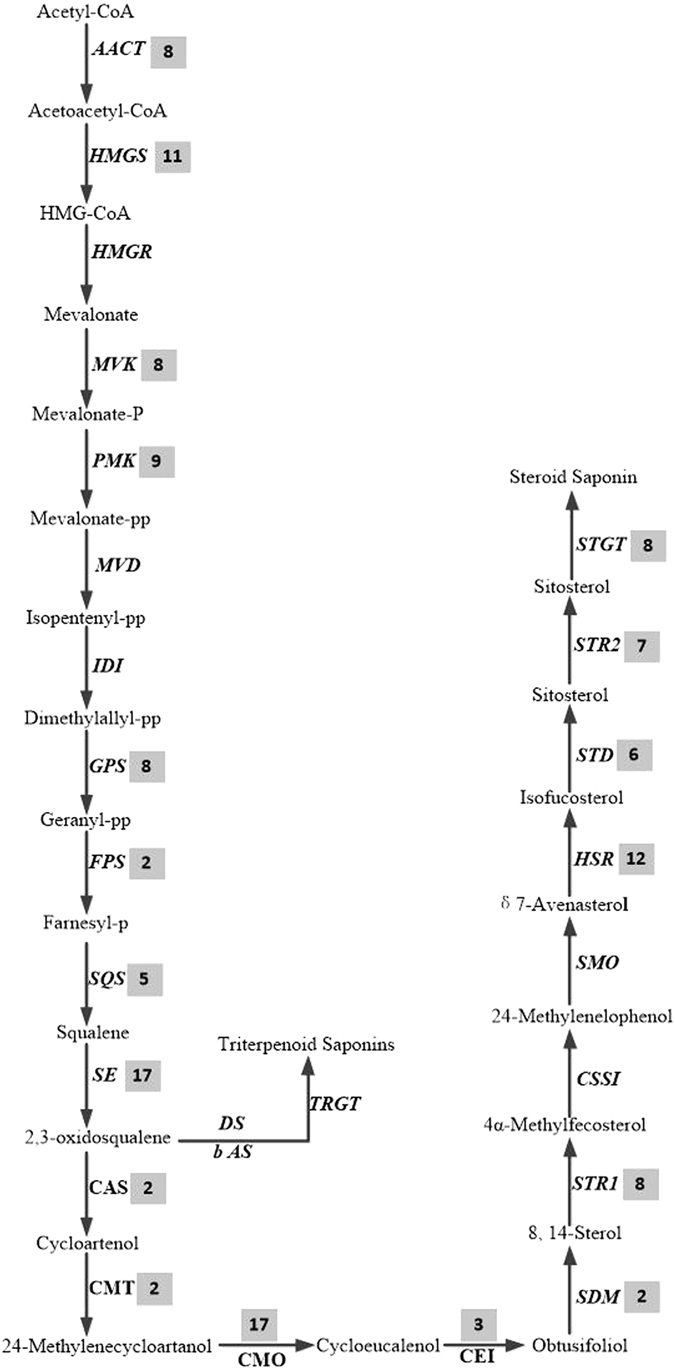
Saponin biosynthetic pathways in ZMD. The number in the grey shaded area is the number of genes encoding the enzyme. Abbreviations: AACT, acetyl-CoA acetyltransferase; HMGS, hydroxymethylglutaryl-CoA synthase; HMGR, hydroxymethylglutaryl-CoA reductase; MVK, mevalonate kinase; PMK, phosphomevalonate kinase; MVD, diphosphomevalonate decarboxylase; IDI, isopentenyl diphosphate isomerase; GPS, geranylgeranyl pyrophosphate synthase; FPS, farnesyl diphosphate synthase; SQS, squalene synthase; SE, squalene monooxygenase/epoxidase; CAS, cycloartenol synthase; CMT, cycloartenol-C-24-methyltransferase; CMO, methylsterol monooxygenase; CEI, cycloeucalenol cycloisomerase; SDM, sterol 14-demethylase; STR1, delta-(14)-sterol reductase; CSSI, cholesterol Delta-isomerase; SMO2, methylsterol oxidase; STD, delta-7-sterol-5-desaturase; HSR, 7-dehydrocholesterol reductase; STR2, delta (24)-sterol reductase; STGT, sterol 3-beta-glucosyltransferase; bAs, beta-amyrin synthase; DS, dammarenediol synthase; GT, glucosyltransferase.

From the SwissProt database, a total of 135 unigenes encoding key enzymes in the putative backbone biosynthesis of saponins were identified. Among these unigenes, 92 and 103 were also annotated using the KEGG and Nr databases, respectively (Table S5). Saponins comprise a large family of structurally related compounds containing a steroid or triterpenoid aglycone (sapogenin) linked to one or more oligosaccharide moieties through a glycosidic linkage. Saponin biosynthesis is also divided into two parts. The upstream pathway is 2,3-oxidosqualene synthesized from acetyl-CoA and catalysed by acetyl-CoA acetyltransferase (AACT), hydroxymethylglutaryl-CoA synthase (HMGS), hydroxymethylglutaryl-CoA reductase (HMGR), mevalonate kinase (MVK), phosphomevalonate kinase (PMK), diphosphomevalonate decarboxylase (MVD), isopentenyl diphosphate isomerase (IDI), geranylgeranyl pyrophosphate synthase (GPS), farnesyl diphosphate synthase (FPS), squalene synthase (SQS) and squalene monooxygenase/epoxidase (SEQ)^17^ (Fig. 10). Sixty-eight unigenes encoding enzymes in the upstream pathway were obtained, including 8 AACT, 11 HMGS, 8 MVK, 9 PMK, 8 GPS, 2 FPS, 5 SQS and 17 SEQ, which are shared with triterpene saponin and steroidal saponin biosynthesis. The downstream pathway includes two primary branches for triterpene saponin and steroidal saponin biosynthesis. The branch of triterpene saponin biosynthesis is distributed primarily in plants from *Araliaceae*, *Leguminosae*, *Cucurbitaceae* and *Polygalaceae*; this biosynthesis is catalysed by beta-amyrin synthase (bAs) or dammarenediol synthase (DS) to synthesize β-amyrin or dammarenediol, which is further modified by glycosyltransferases (GTs)^18^. *O*. *japonicus* tubers produce steroidal saponins, and the downstream branch metabolizes 2,3-oxidosqualene catalysed by cycloartenol synthase (CAS) to biosynthesize cycloartenol. Further modifications occur through the actions of many enzymes, including oxidase, reductase, desaturase, isomerase, methyltransferase and glucosyltransferase^18, 38^. In the downstream pathway, 67 unigenes were identified (Fig. 10). Similar to the flavonoid biosynthesis pathway, most unigenes were significantly down-regulated with increasing growth stage (Fig. 8B). Seven unigenes were selected for validation using quantitative RT-PCR, and the results were consistent with the FPKM values in the expression trends (Fig. 9B and Table S6).

### Identification and expression analysis of unigenes related to polysaccharide biosynthesis

**Figure 11:**
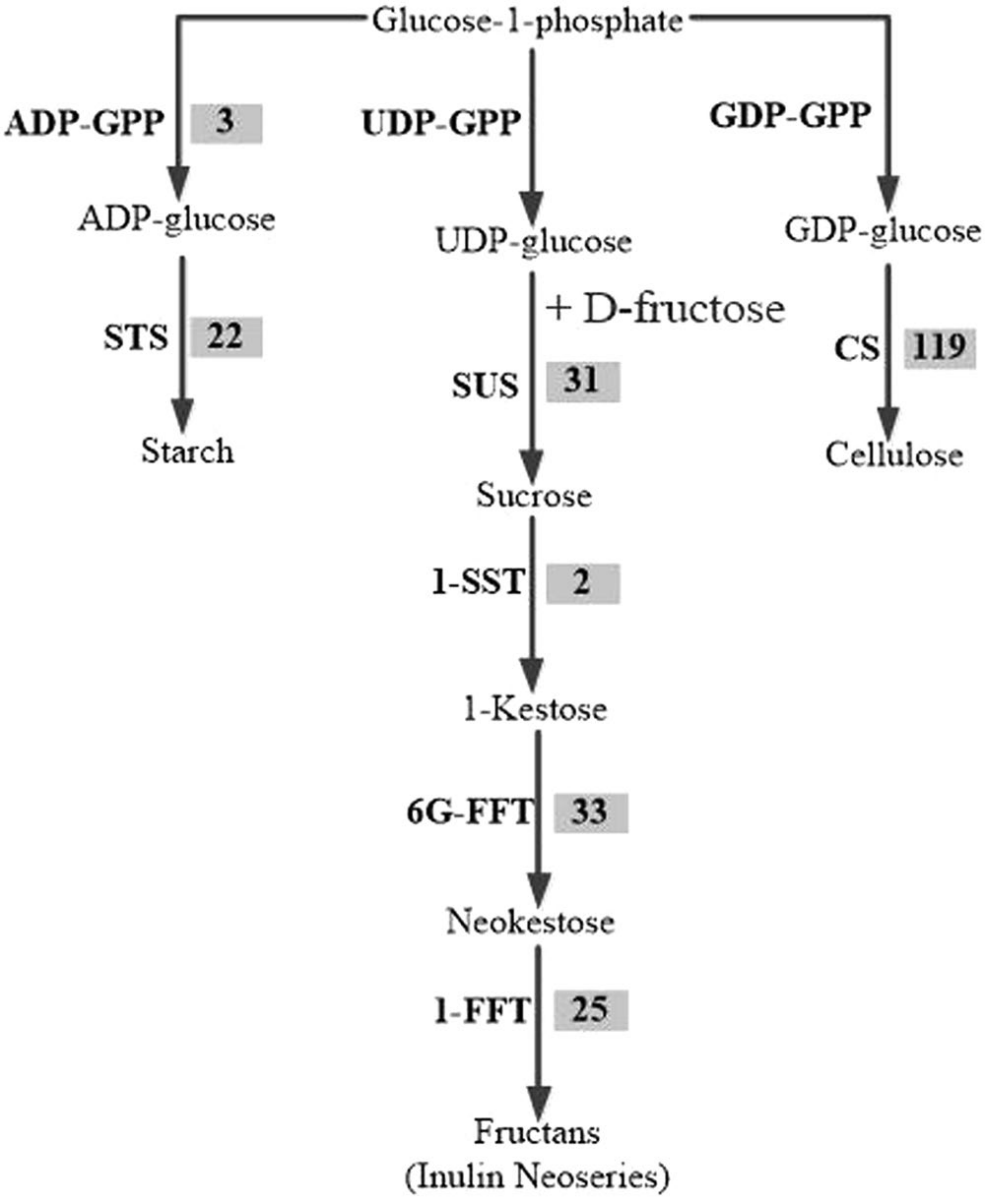
Polysaccharide biosynthetic pathways in ZMD. The number in the grey shaded area is the number of genes encoding the enzyme. Abbreviations: ADP-GPP, ADP-glucose pyrophosphorylase; GDP-GPP, GDP glucose pyrophosphorylase; 6-SFT, sucrose:fructan 6-fructosyltransferase; 1-FFT, fructan:fructan 1-fructosyltransferase; 1-SST, sucrose:sucrose 1-fructosyltransferase; CES, cellulose synthase; SUS, sucrose synthase; STS, starch synthase.

A total of 236 unigenes encoding enzymes for the putative backbone biosynthesis of polysaccharides were identified from the SwissProt database. Among these unigenes, 119 were for cellulose synthase (CS), 59 were for 6(G)-fructosyltransferase (6G-FFT) and fructan 1-fructosyltransferase (1-FFT), 31 were for sucrose synthase (SUS), 22 were for starch synthase (STS), 3 were for ADP-glucose phosphorylase (ADP-GPP) and 2 were for sucrose:sucrose 1-fructosyltransferase (1-SST) (Fig. 11 and Table S5). Unlike the flavonoid and saponin biosynthesis pathways, many expression levels were lower in the first year, and the highest expression levels occurred in the second year (Fig. 8C and Table S5). The polysaccharides contents are rich in *O*. *japonicus* tubers^3^, particularly the contents of fructan, which is an important storage carbohydrate in many plant families. Fructan is a polymer of fructose and is widely distributed in *Liliales* such as *Agave* ^39^, *Asparagus* ^40^, *Leeks* ^41^, *Garlic* ^42^ and *Onions* ^43^. As a soluble food, fructan has beneficial effects on human intestinal microorganisms as a food ingredient^44^, although it cannot be digested by humans. Furthermore, some bifunctional genes were also identified, such as TR7922|c3_g3 and TR16942|c0_g4, which encode the 6G-FFT/1-FFT protein and have a relatively high level of expression compared with other unigenes. The 6G-FFT or 1-FFT protein is an important enzyme in the formation of the inulin neoseries pathway. Inulin is a type of fructan accumulated by *Liliales*, and 6G-FFT can catalyse the transfer of a fructose residue of 1-kestose to the carbon 6 of the glucose moiety of sucrose to form neokestose and then elongate the fructan chain through 1-FFT to form an inulin neoseries^45^. Many studies have found various bioactive polysaccharides in *O*. *japonicus* tubers; for example, MDG-1 is a water-soluble β-D-fructan polysaccharide with an average molecular weight of 3,400 Da that constitutes approximately 30% (w/w) of the tuber of *O*. *japonicus*. MDG-1 contains a backbone composed of fructofuranosyl (Fruf) (2 → 1) and branches of Fruf (2 → 6) and Fruf (2 → 1) with an average of 2.8 main chain residues. The polysaccharide contains trace α-D-Glc and may be neo-inulin type (β (2–1) Fru–Fru linkages); these linkages occur through a biosynthetic process that may be related to 6G-FFT and 1-FFT^46^. Lastly, to confirm that the unigenes from sequencing and computational analysis were indeed expressed, we also analysed the differences in the gene expression profiles in the different growth stages. Four unigenes were selected for validation using quantitative RT-PCR, and the results of were consistent with the FPKM values in the expression trends (Fig. 9C and Table S6).

### Differently aged ZMD are related to flavonoid, saponin and polysaccharide accumulation

**Figure 12:**
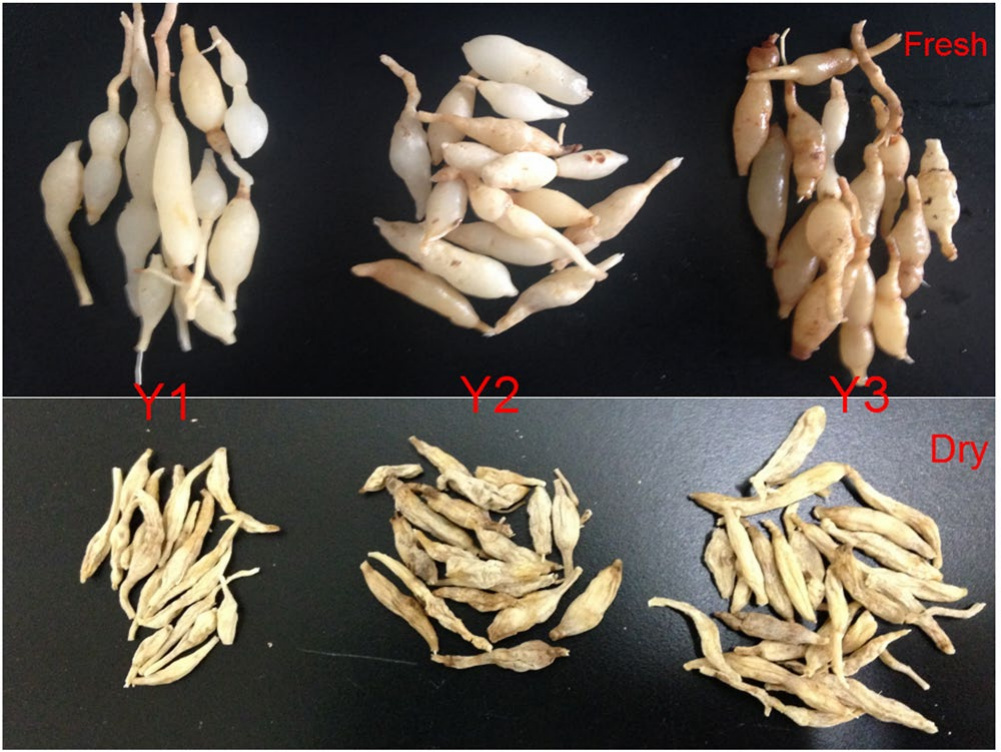
Morphology of the fresh and dry tubers of “ZMD” at three different growth stage. The fresh tubers is shown on the top of the figure, while the dry tubers in shown in the bottom. Abbreviations: Y1, one-year-old ZMD; Y2, two-year-old ZMD; Y3, three-year-old ZMD.

**Figure 13:**
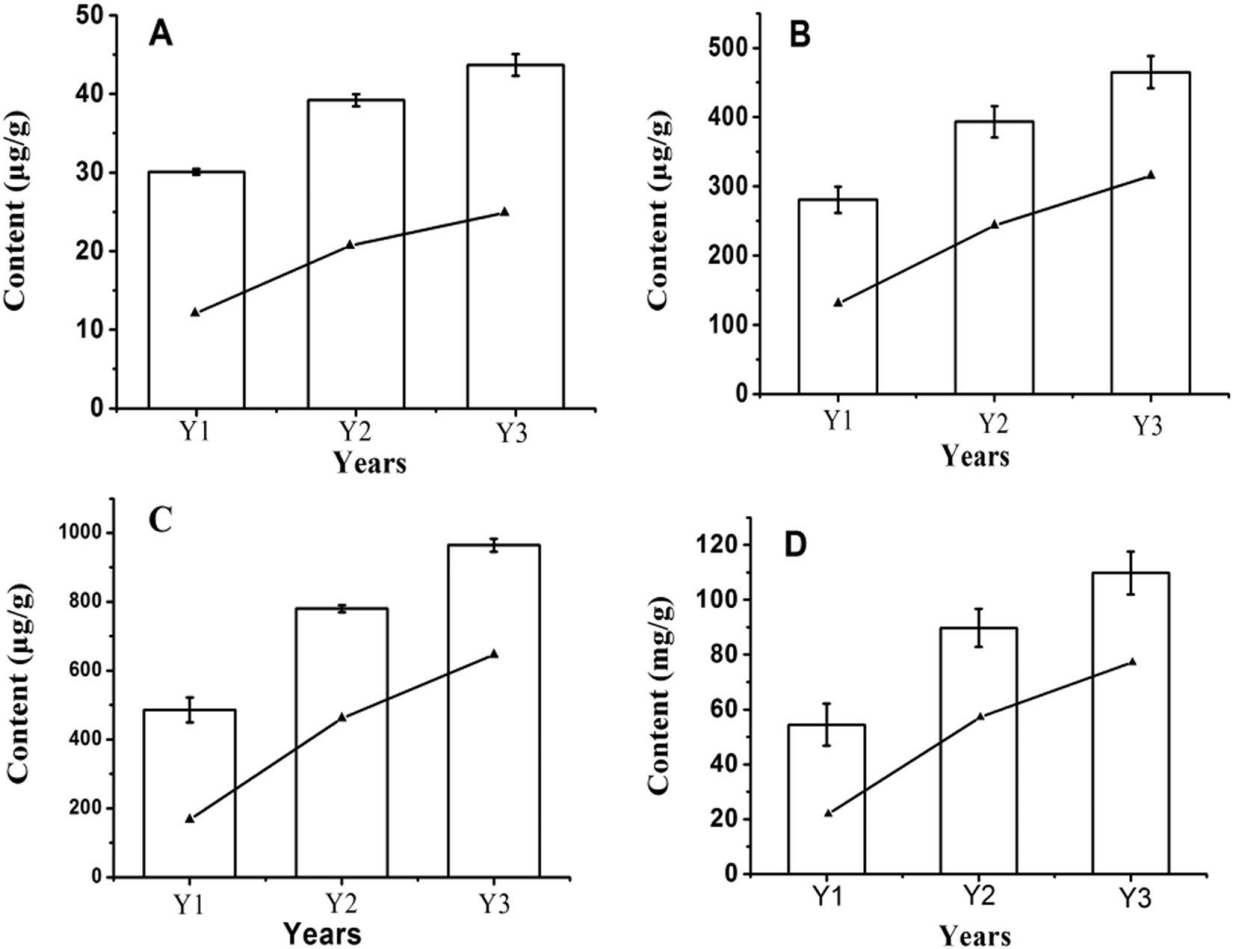
Comparison of the content of different metabolites among the three growth stage. The lines indicate the growth rates. (**A**) Methylophiopogonanone A (ng); (**B**) Total flavonoids (ng); (**C**) Total saponins (ng); (**D**) Total polysaccharides (mg). Data reported represent the average and standard error of the mean of three independent experiments. Abbreviations: Y1, one-year-old ZMD; Y2, two-year-old ZMD; Y3, three-year-old ZMD.

ZMD requires three years from planting to harvest. The morphological characteristics of the tubers of the three growth stages were similar, except for the surface colour. As shown in Fig. 12, the size of fresh tubers at Y1 was the same as that at Y2 or Y3, although fresh tubers at Y1 appeared translucent and contained more water. The fresh tubers at Y2 or Y3 appeared yellow and swollen. After oven drying (70 °C), the weight of Y1 tubers decreased more than that of Y2 or Y3 tubers (Fig. 12). The tuber contents of methylophiopogonanone A, flavonoids, saponins and polysaccharides at the three different growth stages were investigated using HPLC and SP. Equal quantities (5.0 g) of fresh ZMD tubers were used to compare the flavonoid, saponin and polysaccharide contents at the different growth stages. The methylophiopogonanone A content was determined by HPLC, and the total flavonoid, saponin and polysaccharide contents were determined using SP. These contents increased with growth stage, but the accumulation rate decreased with growth stage in ZMD (Fig. 13 and Table S7).

Transcriptome analysis showed that the expression level of genes related to the biosynthesis of flavonoids and saponins decreased with growth stage. The expression of genes related to the biosynthesis of polysaccharides first increased and then decreased with growth stage. However, of note, the three genetic pathways continued to be expressed at Y3 (Fig. 10). Based on these results, flavonoids and saponins were primarily biosynthesized in Y1, polysaccharides were primarily biosynthesized in Y2, and these compounds gradually accumulated with increasing growth stage in ZMD tubers. When these compounds accumulate to a certain level, feedback inhibits further gene expression. Additionally, many studies reveal that primary and secondary metabolite biosynthesis is influenced by TFs such as MYB, bHLH, MADS, GATA and WRKY^47–49^. In this study, 1,508 unigenes encoded TFs that represented more than 70 different families, as annotated by the SwissProt database, including 256 unigenes for the WRKY TF family, 193 unigenes for the ethylene-responsive TF family, 161 unigenes for the bHLH TF family, 80 unigenes for the MYB TF family, 31 unigenes for the MADS TF family and 787 unigenes for other TF families (Table S8). We found that the expression levels for different TFs differed with growth stage, as shown in detail in Table S8.

MicroRNAs (miRNAs), approximately 19–24 nucleotides in length, are important regulators of many physiological and developmental processes, and their mis-expression leads to a variety of defects in plants^50^. Many studies reveal that primary and secondary metabolite biosynthesis is also influenced by microRNAs; this influence is demonstrated by the effects of miR858, miR828, miR156-SPL9 and miR159 on flavonoid biosynthesis in *Arabidopsis thaliana* ^49, 50^ and *Malus* ^51^; the effects of miR5021, miR5293 and miR5163 on saponin biosynthesis in *Panax notoginseng* ^52^; and the effects of miR1862, miR1874–5p and miR530 on carbohydrate metabolism in indica rice^53^. These effects should be investigated in future studies.

Plant tubers can be used for asexual propagation, which is the predominant propagation method for many plants, including *Solanum tuberosum*, *Ipomoea batatas* and *Pueraria lobata*. However, based on many years of cultivation, *O*. *japonicus* tubers cannot be used for asexual propagation and will gradually decay and disappear after the third year. As an additional notable feature, the roots of *O*. *japonicus* must be cut when planting; without the cutting, very few or no tubers will grow. This characteristic may indicate that ZMD tubers are temporary tissues that function in nutrition storage and may explain the decreased gene expression levels when tubers are ripe.

## Conclusions

In this study, we sequenced and characterized the transcriptome of ZMD tubers at three different growth stages (Y1, Y2 and Y3). A total of 96,738 unigenes from 16.4 Gb of sequence data were obtained, and 77,409 unigenes were annotated. A total of 6,473, 7,073 and 1,209 DEGs were identified in the Y2_vs_Y1, Y3_vs_Y1 and Y3_vs_Y2 groups, respectively, and most were related to TFs and metabolic pathways. Expression levels decreased with growth stage. Additionally, based on SwissProt database annotation, 254, 135 and 236 unigenes were identified that were related to the biosynthesis of flavonoids, saponins and polysaccharides, respectively. Notably, most unigenes related to the biosynthesis of flavonoids and saponins were down-regulated with growth stage, and most unigenes related to the biosynthesis of polysaccharides were first up-regulated and then down-regulated. However, of note, the three gene pathways continued to be expressed in the third year. We further analysed the contents of methylophiopogonanone A, total flavonoids, total saponins and total polysaccharides in ZMD tubers using HPLC and SP. The contents of all compounds increased with growth stage. Our study may accelerate the understanding of ambiguous physiological processes and their medicinal value at the molecular level and promote the development of natural medicines and the selection of cultivars with medicinal traits.

## Materials and Methods

### Plant materials and RNA isolation

Samples at three growth stages (five samples at Y1, five samples at Y2 and five samples at Y3) were collected from the same plot of land. Samples were dissected from the roots of each individual and immediately stored in liquid nitrogen after cleaning with germ-free water. Total RNA was isolated from each sample (total of 15 samples) using a Plant RNA Kit (Yuanpinghao, China) and treated with polysaccharide removal reagent PLANTaid (Yuanpinghao, China) according to the manufacturer’s instructions. The quantity and integrity of the total RNA were verified using UV spectrophotometry (Nanodrop 2000), and degradation and contamination were assessed using agarose gels (1%).

### RNA-seq library preparation and sequencing

An Illumina PE library (125 bp) was constructed for the application of Illumina HiSeq-2000 sequencing technology to complete the transcriptome sequencing of *O*. *japonicus* tubers. Briefly, a NEXTflex^TM^ Rapid Directional qRNA-Seq^TM^ Kit (Bio Scientific) was used to build cDNA libraries for the three growth stages. First, equal quantities (2.5 μg) of high-quality RNA from the same sample stage were pooled. Second, the NEXTflex^TM^ RNA fragmentation buffer (Bio scientific, USA) was used to chop the mRNA into short fragments that were then used as templates for the synthesis of the first-strand cDNA with first-strand synthesis primers, first-strand synthesis buffer mix and reverse transcriptase; the second-strand cDNA was synthesized with the NEXTflex^TM^ second-strand synthesis mix. Third, cDNAs were purified with Agencourt AMPure XP (Beckman Coulter) magnetic beads and resolved with resuspension buffer for end repair and adenylation. Unique barcode adapters were applied to each library. Finally, the sequencing library was built by PCR amplification and sequenced on the Illumina HiSeq-2000 platform using paired-end technology. The data were deposited in the Genome Sequence Archive (GSA) database under accession no. PRJCA000324.

### *De novo* assembly and functional annotation

Raw reads were cleaned using Trimmomatic (v0.32)^27^, and clean reads were assembled by Trinity (http://trinityrnaseq.github.io)^54^. The parameters for Trinity were as follows: min kmercov 10, min contig length 200, and run butterfly. All unigenes were annotated against databases such as the NT, NR and SwissProt databases with E-values less than 1e^−5^. Functional annotation by GO terms (http://www.geneontology.org) was analysed with the Blast2GO program (Blast2GO v2.5)^55^, and the final classification of the unigenes was based on these GO slims. Pathway assignments were conducted based on the KEGG database (http://www.genome.jp/kegg). Unigenes were compared with the KEGG database using BLASTx with an E-value of less than 1e-5, and a Perl script was developed to retrieve KO (KEGG Orthology) information from the BLASTx results; we established a pathway correlation between unigenes and the database^56^. To further enrich the pathway annotations, unigenes were submitted to the KEGG Automatic Annotation Server (KAAS), and the single-directional best-hit information method was selected. To identify the enriched pathways, a binormal test was used to measure the relative coverage of the annotated KEGG orthologous groups of a pathway against the background, and the pathways with adjusted p-value < 0.05 were classified as significantly enriched^57, 58^.

### Unigenes of differential expression and qRT-PCR analysis

Unigene expression levels were calculated using the FPKM method. The assembled transcriptome was used as a reference database, and gene expression levels were determined for each sample. Briefly, clean reads were mapped back to the reference transcriptome by Bowtie 2, and the read count for each gene was obtained from the mapping results using RSEM with Default Parameters^59^. The data were normalized for variation in sequencing depth with FPKM, and differential expression analysis was performed using DEGseq (2010)^60^. Q-value < 0.05 and |log_2_ (fold change)| ≥1 defined the threshold for significant differential expression. To confirm the results of the DEG analyses by RNA-seq, the expression levels of 17 unigenes related to the synthesis of flavonoids, saponins and polysaccharides were quantified and compared with the ZMD growth stage using qRT-PCR. First, 1 μg of RNA from each sample was used to reverse transcribe first-strand cDNA using Super Script III reverse transcriptase (Invitrogen) according to the manufacturer’s instructions. Gene-specific primer pairs (Table S6) that were designed using Primer 5.0 were used for real-time PCR. Reactions were performed with SYBR Green PCR Master Mix (Applied Biosystems) in an ABI PRISM 7500 Fast Real-time system. Expression levels of the selected unigenes were normalized to that of tubulin beta-2, the internal reference gene. PCR conditions were as follows: 95 °C for 2 min, followed by 40 cycles of 95 °C for 15 s, 55 °C for 15 s and 72 °C for 30 s. The relative expression was determined using the 2^−ΔCt^ method^14^. All experiments were repeated using five biological and three experimental replicates, and the data were analysed statistically.

### Methylophiopogonanone A, total flavonoid, total saponin and total polysaccharide contents with ZMD year

*O*. *japonicus* was cultivated in the city of Cixi, Zhejiang Province, China; ChiaTaiQingchunbao Pharmaceutical Co. Ltd. managed this zone. We collected 15 samples from three growth stages (five samples each of Y1, Y2 and Y3) from the same plot of land. The samples from the three different ZMD growth stages were washed with running tap water and rinsed with distilled water to remove any adsorbed contaminants from the sample surface. Equal quantities of the three groups of ZMD (each 80 g) were oven dried at 70 °C for 48 h to remove moisture, and each quantity was divided into 16 parts. Each part was ground by mortar into powder, passed through a sieve (20 mesh) and collected for extraction.

The content of methylophiopogonanone A was determined by HPLC-UV and the criterion of methylophiopogonanone A (Chengdu Must Bio-tech. Co., Ltd.; purity >99%**)**. The sample was separated on a Kromasil 100–5 C_18_ column (5 μm, 4.6 × 250 mm, Agilent). The mobile phase consisted of acetonitrile (A) and water containing 0.1% phosphoric acid (B). The gradient programme was 58% A within 0–30 min; the mobile phase flow rate was 1.0 ml/min; a volume of 20 μl was injected into the HPLC instrument for analysis; the detector was monitored at 208 nm; and the column temperature was set at 30 °C. The powder was extracted with 40 ml of 90% (v/v) ethanol in an ultrasonic water bath for 1 h (500 w, 40 KHz, 50 °C) and filtered. The filtrate was evaporated to dryness and dissolved in 10 ml of methanol. The solution was filtered through a 0.45-μm micropore membrane before use. All experiments were repeated using three biological replicates. Data were analysed statistically, and the analyses were performed on an Agilent series 1200 HPLC instrument (Agilent, Waldbronn, Germany).

The total flavonoid content was determined using the aluminium nitrate colourimetric method and the criterion of rutin^61^ (National Institute for the Control of Pharmaceutical and Biological Products, purity = 92.5%). Specifically, the above extracts (3.5 ml) were added to test tubes and mixed with 0.5 ml of 5% sodium nitrite, left to stand for six minutes at room temperature and then mixed with 0.5 ml of 10% aluminium nitrate and left to stand for an additional six minutes at room temperature; lastly, the samples were mixed with 5 ml of 1% sodium hydroxide and 0.5 ml of methanol. After standing for 12 min at room temperature, the absorbance value of the reaction mixtures was measured at 510 nm. All experiments were repeated using three biological and three experimental replicates, and the data were analysed statistically.

The total saponin content was determined using the perchloric acid method and the criterion of ruscogen^62^ (National Institute for the Control of Pharmaceutical and Biological Products, purity = 98.4%). The powder was extracted with 50 ml of methanol under an ultrasonic water bath for 1 h (500 w, 40 KHz, 50 °C) and then filtered. Then, 25 ml of filtrate was dried and redissolved in 10 ml of water. Lastly, 10 ml of n-butanol was used to extract the aqueous solution four times, and extracts were combined and dried (total 40 ml). The crystal was dissolved in 25 ml of methanol. The detection steps were as follows: the purified extracts (3 ml) were added to test tubes and left in a hot water bath to dry; 10 ml of perchloric acid was then added, and the plate was kept in a static water bath for 15 min at 95 °C. After cooling to room temperature for 5 min in another water bath, each tube was wiped dry, and ***A***_397 nm_ was measured. All experiments were repeated using three biological and three experimental replicates, and the data were analysed statistically.

The total polysaccharide content was detected using the phenol-sulfuric acid method and the glucose criterion^63^. Many studies find that most polysaccharides are insoluble in concentrated ethanol, so we dissolved the residue (with filter paper) in 40 ml of water under an ultrasonic water bath for 1 h (500 w, 40 KHz, 60 °C) and then filtered the liquid. The filtrate was evaporated and diluted with water to 5 L, and 1 ml was collected for the phenol-sulfuric acid method of analysis. All experiments were repeated using three biological and three experimental replicates, and the data were analysed statistically. In detail, 1 ml of extracts in a test tube was added rapidly to 5 ml of concentrated sulfuric acid. Immediately, 1 ml of 5% phenol was added, and the plate was kept in a static water bath for 15 min at 95 °C. After cooling to room temperature for 5 min in another water bath, each tube was wiped dry, and ***A***_490 nm_ was measured.

## Electronic supplementary material

Table S1

Table S2

Table S3

Table S4

Table S5

Table S6

Table S7

Table S8

Supplementary Figures
