# Supplementary material for: De novo assembly and annotation of the Zhe-Maidong (Ophiopogon japonicus (L.f.) Ker-Gawl) transcriptome in different growth stages
Source: Sci Rep. 2017 Jun 15;7:3616. doi: 10.1038/s41598-017-03937-w (PMC5472570; doi:10.1038/s41598-017-03937-w)
Supplement: Supplementary file 9 — Supplementary Figures [file 41598_2017_3937_MOESM9_ESM.pdf]

Supplementary Information

De novo assembly and annotation of the Zhe-Maidong (_Ophiopogon japonicus_ (L.f.) Ker-Gawl) transcriptome in different growth stages

HuijunLiu[1], Ying Wang[2,3], Tingzhang Wang[2,3], Xuhui Ying[6], Rongrong Wu[4,5,*], Huan Chen[1,2,3,*]

[1]College of Pharmaceutical Science, Zhejiang University of Technology, Hangzhou 310014, China

[2]Zhejiang Institute of Microbiology, Hangzhou 310012, China

[3]Key laboratory of microbial technology and bioinformatics of Zhejiang Province, Hangzhou 310012, China

[4]Department of Cardiology, Second Affiliated Hospital, College of Medicine, Zhejiang University, Hangzhou, 310009, China

[5]Cardiovascular Key Laboratory of Zhejiang Province, Hangzhou, 310009, China

[6]ChiaTaiQingchunbao Pharmaceutical Co., Ltd

*To whom correspondence should be addressed.

Huan Chen, E-mail address: chenhuan7809@gmail.com;

Rongrong Wu, E-mail address:realagatha@zju.edu.cn

Supplementary Figures

Figure S1 Three expression patterns were classified into biological process based on gene oncology(GO). Abbreviations: Up-down(red bar), genes with expression first increased and then decreased pattern; Down(green bar), genes with decreased expression pattern; Up(blue bar), genes with increased expression pattern.

Figure S2 Three expression patterns were classified into cellular component based on gene oncology(GO). Abbreviations: Up-down(red bar), genes with expression first increased and then decreased pattern; Down(green bar), genes with decreased expression pattern; Up(blue bar), genes with increased expression pattern.

Figure S3 Three expression patterns were classified into molecular function based on gene oncology(GO). Abbreviations: Up-down(red bar), genes with expression first increased and then decreased pattern; Down(green bar), genes with decreased expression pattern; Up(blue bar), genes with increased expression pattern.

Figure S1

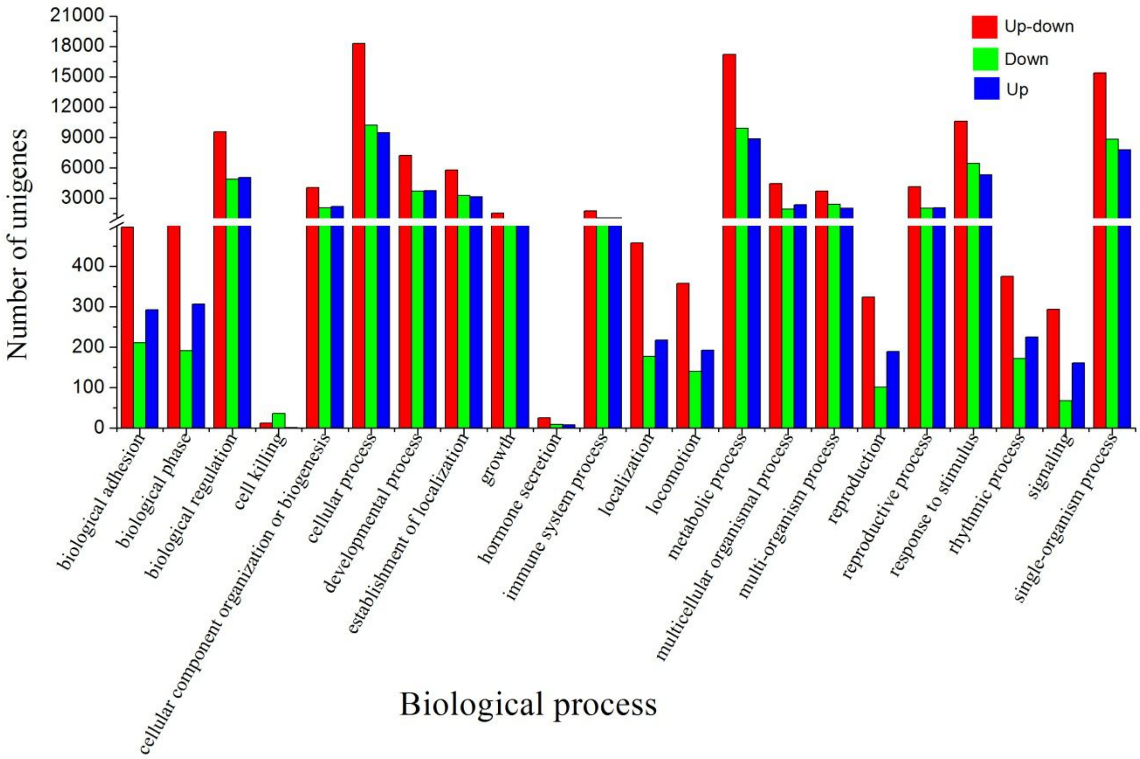

Figure S1 Three expression patterns were classified into biological process based on gene oncology(GO). Abbreviations: Up-down(red bar), genes with expression first increased and then decreased pattern; Down(green bar), genes with decreased expression pattern; Up(blue bar), genes with increased expression pattern.

Figure S2

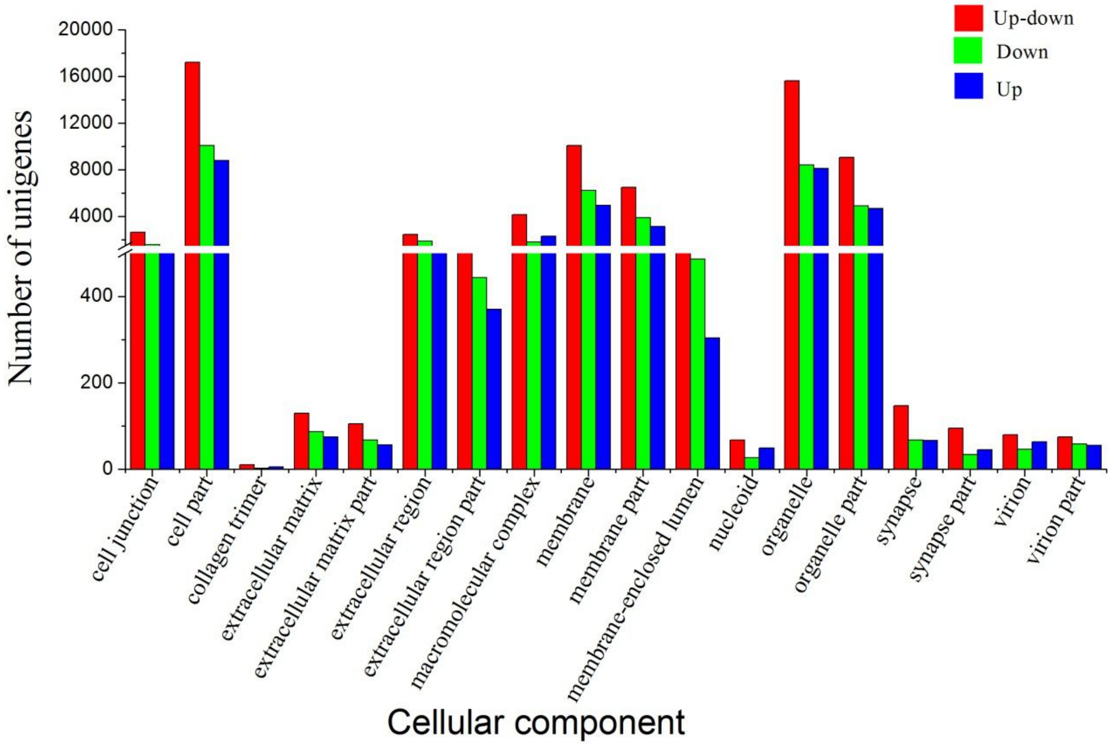

Figure S2 Three expression patterns were classified into cellular component based on gene oncology(GO). Abbreviations: Up-down(red bar), genes with expression first increased and then decreased pattern; Down(green bar), genes with decreased expression pattern; Up(blue bar), genes with increased expression pattern.

Figure S3

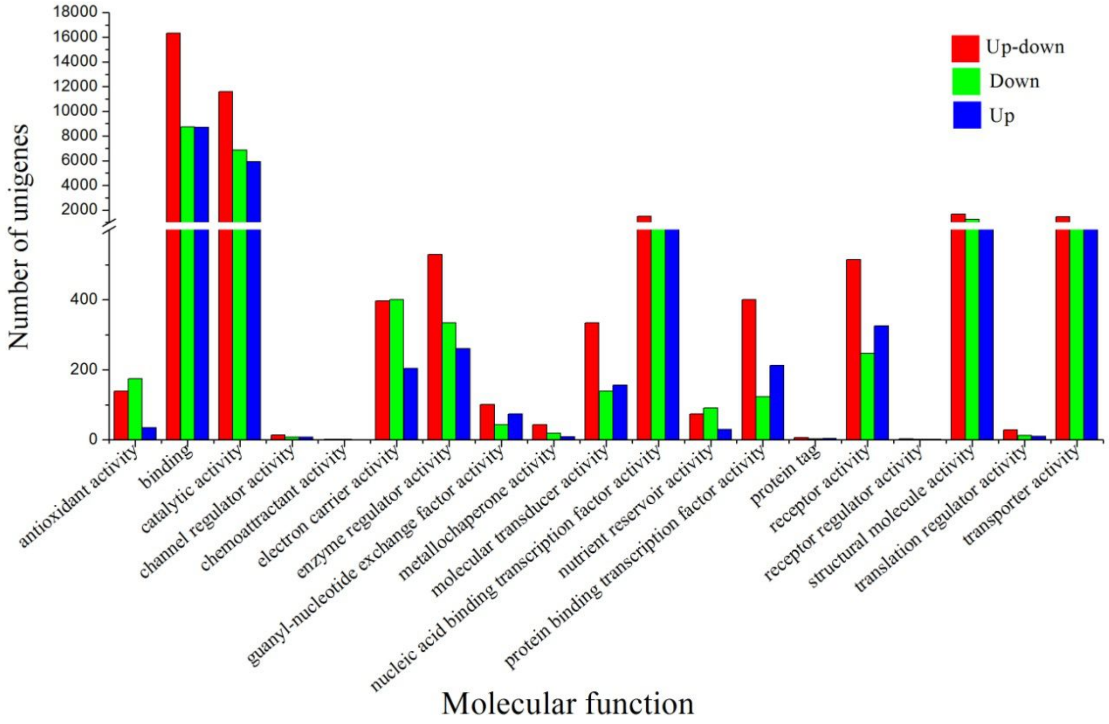

Figure S3 Three expression patterns were classified into molecular function based on gene oncology(GO). Abbreviations: Up-down(red bar), genes with expression first increased and then decreased pattern; Down(green bar), genes with decreased expression pattern; Up(blue bar), genes with increased expression pattern.
